# The Fall Armyworm and Larger Grain Borer Pest Invasions in Africa: Drivers, Impacts and Implications for Food Systems

**DOI:** 10.3390/biology13030160

**Published:** 2024-02-29

**Authors:** Shaw Mlambo, Macdonald Mubayiwa, Vimbai L. Tarusikirwa, Honest Machekano, Brighton M. Mvumi, Casper Nyamukondiwa

**Affiliations:** 1 Department of Biological Sciences and Biotechnology, Botswana International University of Science and Technology, Private Bag 16, Palapye 10071, Botswana; shawmlambo@gmail.com (S.M.); mmubayiwa@gmail.com (M.M.); 2 Department of Biology, The University of Western Ontario, London, ON N6A 5B7, Canada; vtarusik@uwo.ca; 3 Department of Zoology and Entomology, University of Pretoria, Private Bag X20, Pretoria 0028, South Africa; honest.machekano@up.ac.za; 4 Department of Agricultural and Biosystems Engineering, University of Zimbabwe, Mount Pleasant, Harare P.O. Box MP167, Zimbabwe; mvumibm@agric.uz.ac.zw; 5 Department of Zoology and Entomology, Rhodes University, Makhanda 6140, South Africa

**Keywords:** alien pest invasions, food and nutrition security, invasive insect species, *Prostephanus truncatus*, *Spodoptera frugiperda*

## Abstract

**Simple Summary:**

The fall armyworm and larger grain borer are two of the main invasive cereal insect pests of cereal crops in Africa. These pests cause successive synergistic damage to maize in the field and after harvesting, resulting in huge food losses in Africa. The two invaders share invasive characteristics such as a high reproductive capacity, high thermal tolerance, pesticide resistance and the ability to feed on numerous hosts—traits that help them outcompete native species. Along with these characteristics, climate change, increased anthropogenic activities and factors such as the lack and/or poor tracking of natural enemies in the case of larger grain borer and an increase in mixed cropping under smallholder farming systems enabling host-switching in the case of the fall armyworm, have inevitably facilitated the continental spread of the two pests. Cumulative losses from the successive attack of the two insect pests suggest that 30–100% food losses are incurred when the two co-exist within the same environment. Improved management and containment of the fall armyworm and larger grain borer through the curtailment of plant material smuggling, improved phytosanitary regulations, public awareness and integrated pest management strategies can contribute towards improving food and nutrition security in Africa.

**Abstract:**

Invasive alien species (IAS) are a major biosecurity threat affecting globalisation and the international trade of agricultural products and natural ecosystems. In recent decades, for example, field crop and postharvest grain insect pests have independently accounted for a significant decline in food quantity and quality. Nevertheless, how their interaction and cumulative effects along the ever-evolving field production to postharvest continuum contribute towards food insecurity remain scant in the literature. To address this within the context of Africa, we focus on the fall armyworm, *Spodoptera frugiperda* (J.E. Smith) (Lepidoptera: Noctuidae), and the larger grain borer, *Prostephanus truncatus* (Horn) (Coleoptera: Bostrichidae), two of the most important field and postharvest IAS, respectively, that have invaded Africa. Both insect pests have shown high invasion success, managing to establish themselves in >50% of the African continent within a decade post-introduction. The successive and summative nature of field and postharvest damage by invasive insect pests on the same crop along its value chain results in exacerbated food losses. This systematic review assesses the drivers, impacts and management of the fall armyworm and larger grain borer and their effects on food systems in Africa. Interrogating these issues is important in early warning systems, holistic management of IAS, maintenance of integral food systems in Africa and the development of effective management strategies.

## 1. Introduction

Biological invasion is the introduction, establishment, spread and proliferation of biological organisms outside their native range [1]. This introduction and establishment often lead to the reorganisation of ecosystem structures to new ecological equilibria which often affects local biodiversity and ecosystem function [2,3,4]. The United Nations Sustainable Development Goals (SDGs) 2 (“*zero hunger*”) and 12 (“*responsible consumption and production*”) emphasise the attainment of resilient food systems through sustainable production and consumption [5]. However, the maintenance of these vulnerable food systems in Africa has been retarded by climate change, anthropogenic effects [6,7] and associated consequences, such as increased biosecurity threats posed by biological invasions [8]. Invasive insect pests have extensive economic, social and environmental consequences, thus they disproportionally threaten food and livelihood systems, particularly in low- and medium-income countries in Africa [9,10,11]. Increased global connectivity, anthropogenic climate change and a surge in the human population size have accelerated the rate of biological invasions with no indications of imminent saturation [1,12,13,14]. The SDG 12 emphasises improved and sustainable production (improved yields) reinforced by sustainable consumption and even sets specific targets related to the reduction of food loss and waste, including postharvest management [15,16,17,18].

Pests can cost billions of dollars in agricultural losses and control programs and have lasting effects on human populations [13,19,20]. Insect pests, in particular, are also major contributors to the loss of business, export markets and product value and quality [9]. On a global scale, it is estimated that invasive alien species have caused economic losses of at least USD 1.288 trillion (for the period 1970–2017) [13,21]. Global losses incurred from crop damage and efforts directed at pest management are estimated to be USD 76 billion annually [20], whereas those from Africa cumulatively ranged between USD 18.2 billion and USD 78.9 billion between 1970 and 2020 [13]. In recent decades, agricultural production in Africa has been severely hampered by invasive insect pests [22] with significant food losses of up to 30% being reported [18,23,24]. In order to meet the food requirements of the exponentially growing human population in Africa, projections suggest that agricultural production must double by the year 2050 [25,26]. However, this doubling of production may compromise sustainability, ecosystems and ecosystem services [27]. For example, invasion by alien insects with a high pest status has had devastating effects on the production of staple cereal crops such as maize and sorghum in Africa [22]. The fall armyworm (FAW), *Spodoptera frugiperda* (J.E. Smith) (Lepidoptera: Noctuidae), is one of the major pests affecting maize and sorghum field production in Africa [13]. On the other hand, the larger grain borer (LGB), *Prostephanus truncatus* (Horn) (Coleoptera: Bostrichidae), is a notorious alien insect pest of stored maize and dried cassava roots which is rapidly spreading in Africa [28,29,30]. The arrival of *P. truncatus* in Africa has doubled maize grain losses in affected areas [31].

**Figure 1:**
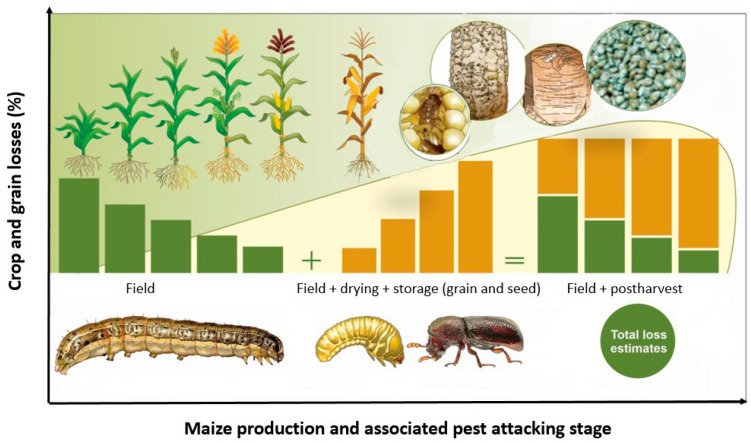
Conceptual hypothetical framework showing cumulative losses associated with *S. frugiperda* damage on field maize and *P. truncatus* damage to stored maize (not drawn to scale; source: Authors). Crop damage from *S. frugiperda* is usually higher during initial crop growth stages and declines as the crop approaches physiological maturity at which stage *P. truncatus* takes over up to postharvest storage, thus inflicting cumulative synergistic losses that can be monetarily quantified. However, no scientific data are available to validate our hypotheses as yet.

Reported maize field losses from *S. frugiperda* range from 9 to 54% in Africa [32,33] while those of *P. truncatus* range from 20 to 50% on the weight basis reported within 6–9 months of storage [34,35,36,37]. Combined, therefore, *S. frugiperda* and *P. truncatus* may account for between 30 and 100% in food losses where they successively attack the same crop(s) along the different stages of the production chain under the same farmer. *Spodoptera frugiperda* larvae damage maize at all stages of growth, including cobs, though it is most devastating during early crop growth phases. Field losses are thus higher during early maize growth phases and decrease during late growth and physiological maturity stages (Figure 1). *Prostephanus truncatus*, on the other hand, infests maize cobs at physiological maturity and persists during grain drying to the storage phases [38]. Grain and seed losses due to both larvae and adult stages increase with increasing storage duration (Figure 1). When the two insect pests occur successively in the same niche, the cumulative field losses due to *S. frugiperda* in the field plus drying and storage losses due to *P. truncatus* are thus higher, resulting in an excessive loss impact per farmer.

**Figure 2:**
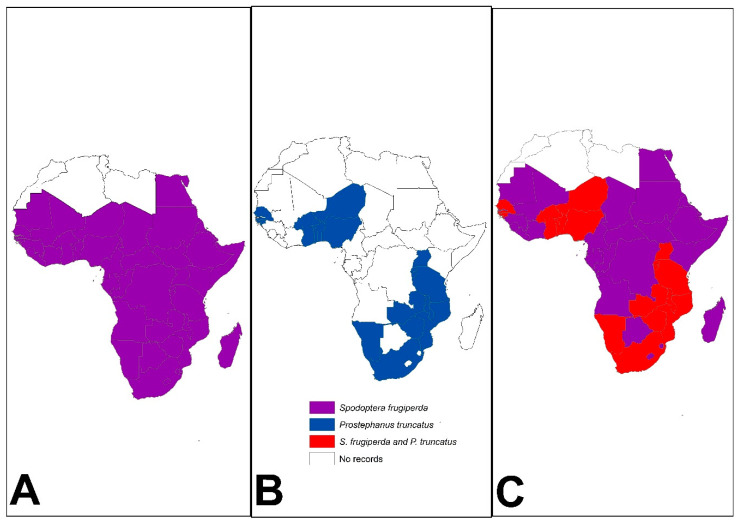
Distribution of (**A**) *Spodoptera frugiperda* (see [39] for distribution time scale) and (**B**) *Prostephanus truncatus* in Africa (see [30] for distribution time scale) as of July 2023. Insert (**C**) shows countries where both pests have been reported (Source: Authors’ compilations from various sources).

In less than 10 years since its first detection in West and Central Africa in 2016, *S. frugiperda* had spread to 47 out of 54 African countries (Figure 2), causing significant food and nutrition threats [39,40]. The pest prefers maize and sorghum, although it can feed and complete its life cycle on >350 plant species, including several food crops [39,41]. This polyphagous characteristic enables the pest to survive across diverse host environments. *Spodoptera frugiperda* larvae defoliate crops during vegetative growth reducing crop growth and reproductive capacity [10,42,43,44,45,46,47]. In maize, the pest attacks the crop up to the soft dough stage, increasing the crop’s vulnerability to additive losses through storage insects and mycotoxin contamination [45]. *Spodoptera frugiperda* is multivoltine, facilitating a high and quick population build-up. Adult females can lay ~300 eggs on the underside of leaf sheaths [48]. The first and second instars can disperse by suspending themselves on silk threads and are swung by the wind to reach other host plants [40].

On the other hand, *P. truncatus* has increased the magnitude of postharvest losses incurred in stored maize and dried cassava roots in Africa due to its characteristic extensive tunnelling and feeding that reduces whole kernel grains and cassava chips into powder [49,50,51]. The pest was first reported in Tanzania in 1981 [52,53] and has since spread to at least 21 African countries in the last four decades (Figure 2) in sub-Saharan Africa [30,31,54]. *Prostephanus truncatus* can also survive on wild hosts in the forest [55,56,57] and exhibits a sporadic attack, making it difficult to manage [49,50,55,58]. Increased feeding behaviour has been observed at higher temperatures [58]. The adult beetles can disperse through flight in search of food and suitable oviposition sites [49,59]. Males release an aggregation pheromone attractive to both sexes when they encounter a favourable host, and this allows the beetles to quickly colonise and exploit host resources [49]. The beetle can burrow through hard material and prefers the bottom of bagged or bulk grain for leverage [60] or maize cobs [61].

Regardless of the overwhelming evidence that *S. frugiperda* and *P. truncatus* are the main field and postharvest pests of staple maize in Africa [34,62], their effects on food systems have often been studied independently, even when they occupy the same ecological niche; see, e.g., [31,42,44,63]. However, their successional attack on the different stages of maize, for example, shows that these two pests may have devastating negative complementary and interactive effects that represent a damage and loss continuum against food systems. Here, we thus interrogate the food systems threats in Africa posed by *S. frugiperda* and *P. truncatus*, with special reference to impact on the maize crop cycle to demonstrate how the pest additive interactions through successional damage may exacerbate food losses. The objectives of this review were thus to document: (i) the main maize invasive pests as the key drivers of food loss in Africa, (ii) the drivers of pest invasions in Africa, (iii) the invasive characteristics of *S. frugiperda* and *P. truncatus*; (iv) the economic and ecological costs of *S. frugiperda* and *P. truncatus* as IAS; and (v) the national and continental management strategies for pest invasions. Such insights could help in assessing efficacious management options for pest invasions, improve the resilience and integrity of food production systems, consequently safeguarding food and nutrition security in Africa in response to SDGs 2 and 12.

We conducted a systematic literature search using different key words (including *S. frugiperda* in Africa, economic costs of *S. frugiperda*, damage due to *S. frugiperda*, *P. truncatus* in Africa, damage due to *P. truncatus*) and search engines such as Google Scholar, Web of Science, Google, JSTOR Search and Scopus [64,65]. We then used Mendeley to organise the downloaded articles and manage citations [66]. We first gave a background of food security vulnerability in Africa; second, we elucidated the potential drivers of biological invasions in Africa (emphasising the role of *S. frugiperda* and *P. truncatus*); third, the economic and ecological consequences of these biological invasions to local economies and food security in general; and finally, we discuss possible management strategies for these biological invasions.

In the literature, crop pests have often been dealt with within the rigid framework of their host crop damage based on the alignment between crop and insect phenology. For example, both *S. frugiperda* and *P. truncatus* damage maize at different phases of the crop’s life cycle, but most studies have dealt with *S. frugiperda* individually within the maize field production phase, while *P. truncatus* has been studied individually within the limits of the postharvest phase. Thus, the respective economic loss impacts have been kept separate, although both impacts are on the same crop and experienced by the same farmer. Although the two pests damage maize at different stages of its production cycle, the underlying factor is the loss to the farmer and nation, and the cumulative impact of the loss to the farmer at these different phases of the production cycle (pre- and post-harvest). The invasive insect pests multiply the loss impact that is experienced by the same grower. When the impact of invasive pests on vulnerable farming communities is analysed through the total loss impact lens, it provides a more realistic representation of the socio-economic and food and nutrition security impact of insect pest invasion in Africa. In recent times, food and nutrition security has been subjected to high biosecurity threats from invasive pests driven by climate change [67].

## 2. Vulnerability of Food Systems in Africa

Significant increase in food production in Asia, Latin America, the Pacific and the Caribbean has been realised in the recent past, leaving Africa and south Asia with the highest concentration of food insecurity in the world [68]. The vulnerability of African food systems may partly emanate from the ever-increasing abiotic and biotic shocks.

### 2.1. Abiotic Factors and Their Effect on Food Systems in Africa

Approximately 70% of African livelihoods are directly dependent on agriculture [69]. However, most croplands in Africa are characterised by poor and declining soil fertility [70] primarily due to long-term monocropping, especially under conventional tillage, removal of crop residues and the lack of external nutrient inputs [71]. Consequently, degraded soils are less responsive to inorganic soil amendments such as mineral fertilisers, and hence, poor maize crop yields ranging from 0.5 to 1.2 t ha^−1^ are reported: way below the potential for maize hybrids [72]. Furthermore, changes in rainfall intensity and distribution patterns, as well as temperature increases, are the major abiotic factors affecting rain-fed agriculture in Africa [69,73]. Temperatures in Africa are expected to rise by approximately 2.6 °C by 2050 if climate change mitigation fails [74]. This will result in reduced surface and groundwater resources [75]. Extreme weather events such as heat waves, droughts, floods and cyclones are also expected to increase [76,77,78,79]. This will have direct impacts on crop yields, food prices and livelihoods [75].

On the contrary, farmers are poorly resourced to adapt to their harsh and changing environments. For example, most African farmers use retained seed owing to high costs and limited access to certified seed [80,81]. Due to poor storage techniques and facilities, retained seed is usually attacked by storage insects, leading to low germinability, low plant vigour, poor crop stands and, consequently, a low yield [81,82,83]. Smallholder communities usually rely on agricultural extensification, where vast lands are cleared to enable agricultural production [82,84]. Extensive agricultural production can lead to deforestation, soil depletion and degradation. Furthermore, it can contaminate underground water resources through other agricultural inputs (e.g., agrochemicals) including other commercial activities such as mining [85,86]. This adds to other negative environmental effects such as harm to non-target organisms and biodiversity losses [87,88]. With the increasing human population, land is continually becoming scarce to support such extensification systems. The majority of smallholder farmers in Africa also have limited access to the requisite information, tools and technologies for insect pest identification and the financial means of managing invasive insect pests [8,45]. Coupled with the pressures of increasing human populations, this often offsets the balance between food production and demand [68]. Biological invasions therefore represent an additional stress to an already burdened and fragile agricultural food system.

### 2.2. Biotic Factors and Their Effect on Food Systems in Africa

Major biotic factors increasing the vulnerability of African agriculture relate to increased pest pressure in agricultural environments. Crop weeds and insect pressure are increasing due to climate- and anthropogenic-related changes. Range expansion and/or the survival of insect pests are increasing owing to altered insect physiology and behaviour, as well as interactions within specific habitats [89]. Insects, being poikilothermic, depend on environmental temperatures for their development and survival [90]. Insect pests are thus expected to have more generations and higher functional responses, hence increased crop damage with climate warming [77] during both production and postharvest storage. This will likely increase the associated cost of control and the losses [22].

## 3. Biological Invasions: Donors, Drivers and Processes Involved

### 3.1. Biological Invasions

Biological invasions involve the successful introduction, establishment and range expansion of a species in a non-native habitat, usually anthropogenically mediated [20,91]. Invasion records started approximately 6000 BC with the unintentional introduction of insect pests of stored grain such as *Sitophilus granarius* L. (Coleoptera: Curculionidae) and *Tribolium confusum* Jacquelin Du Val (Coleoptera; Tenebrionidae) from Eastern to Western Europe [92]. Invasive insect pests have been introduced primarily through interventions aimed at helping local situations (e.g., disaster response) and/or through escape from native ranges [93]. Rarely, insect pests are introduced as contaminants of related commodities [93]. Due to their small sizes, insects are insidious and easily transported into new environments undetected through human activities [94]. Smuggling also plays a significant role in IAS introduction and remains one of the most common methods through which alien insect pests have been introduced, especially into developing countries where phytosanitary measures are still a major concern at ports of entry [95]. Deliberate smuggling of agricultural materials, such as seed, has been reported to have resulted in the introduction of various insect pests of stored maize grain in various regions [95]. In addition, the increased global connectedness and trade routes across both oceans and continents has also become the primary source of IAS introductions [96,97]. In particular, shipping, which accounts for 80% of global trade is believed to account for most biological invasions [97]. While several species and/or numbers may be introduced through transportation, only a few pass through all filters and become invasive [98]. Similarly, the invasion process may also be delayed owing to the ‘lag phase’, that facilitates population build-up and local adaptation before spreading [99] The development of regional and global trade agreements also increases the movement and exchange of commodities, which lead to an increase in the introduction of invasive species into new settings either as contaminants or hitchhikers [100]. To become invasive, organisms must overcome biogeographical barriers due to deliberate or accidental human actions and are able to spread rapidly to colonise new territories in the introduced region [12]. The framework for biological invasions has been well explained by Blackburn et al. [98] and involves transportation, introduction, establishment and spread.

As an invader, the advantages of *S. frugiperda* over native species are pivotal in its establishment. *Spodoptera frugiperda* was first detected on maize in Nigeria and São Tomé and Príncipe in 2016 [62]. The pest has spread across the African continent at alarming speed and is now near omnipresent across the continent [47]. The adult moth can self-disperse by flying over long distances to new environments. The presence of *S. frugiperda* in Egypt, for example, means southern Europe is at risk of invasion as the adult moth can cover more than 500 km of flight in a single generation [101]. In this regard, the top six countries at risk of invasion are Spain (39.1%), Italy (32.2%), Turkey (8.9%), France (6.8%), Greece (5.8%) and Portugal (5.1%), and their aggregated risk of invasion is 97.8% [101]. The insect has high fecundity and a short life cycle which enhances its chances of survival. Additionally, *S. frugiperda* does not diapause, but migrates to warmer environments during winter [39]. Furthermore, the insect can survive on a wide range of hosts other than the preferred maize and rice though the number of generations and individual strengths may be compromised [41]. Due to their high fecundity, insects are more likely to survive and spread quickly to newly introduced environments [102]. There are various modes of dispersal of insects to new environments. These include, but are not limited to, self-dispersal through adult flights, silking (in the case of *S. frugiperda*) and as ‘stowaway baggage’ [98]. For *P. truncatus*, lack of, and/or failure of its natural enemies in invaded areas [103,104] and transportation of infested material (maize grain and dried cassava roots or empty bags) [105], as demonstrated by the enemy release hypothesis [106,107,108], resulted in unregulated populations, wide dispersal and the fast colonisation of hosts leading to high losses in maize and cassava [29]. *Prostephanus truncatus* was first introduced in Africa in Tanzania and Togo [28,52,53] as a pest in imported maize grain [29,54]. At the time of the accidental introduction of *P. truncatus* into Tanzania, there were no suitable pesticides registered for its control as it required organophosphate–pyrethroid combinations rather than just the already available organophosphates which could effectively control all other storage insect pests [109].

### 3.2. ‘Donors’ of Biological Invasions

Though there is no consensus on the precise origin of IAS, it is widely accepted that the area of origin of pests corresponds to the centre of origin of the crops with which they are associated [92]. China and the United States are touted as the major ‘donors’ of invasive crop insects due to the massive agricultural production in these countries [9]. It is also speculated that species from the Northern Hemisphere are better competitors and consequently more effective invaders than those from the Southern Hemisphere [110,111], potentially owing to the climate variability hypothesis [112]. *Spodoptera frugiperda* and *P. truncatus* are known to have originated from the tropical and sub-tropical regions of ‘donor’ meso-America and arrived in Africa in 2016 and the late 1970s, respectively [45,113]. To date, *P. truncatus* has been reported in at least 21 countries [30,54], while *S. frugiperda* has been reported in 47 countries [45,47] across the African continent. *Spodoptera frugiperda*’s invasion of Africa has been more rapid than *P. truncatus,* which arrived earlier but has not been reported in as many countries as the former, implying that *S. frugiperda* is more invasive than *P. truncatus.* However, we acknowledge that there could be other factors at play. For example, to our knowledge, *S. frugiperda*, being a field pest, easily attracts attention from scientists and other stakeholders whereas grain storage tends to be ’hidden’ from the public eye. Similarly, *S. frugiperda* invasion and spread also coincided with the boom in social media and the digital age which may have facilitated its faster publicity relative to the timing of *P. truncatus* invasion and spread.

### 3.3. Drivers of Biological Invasions

#### 3.3.1. Anthropogenic Activities

**Figure 3:**
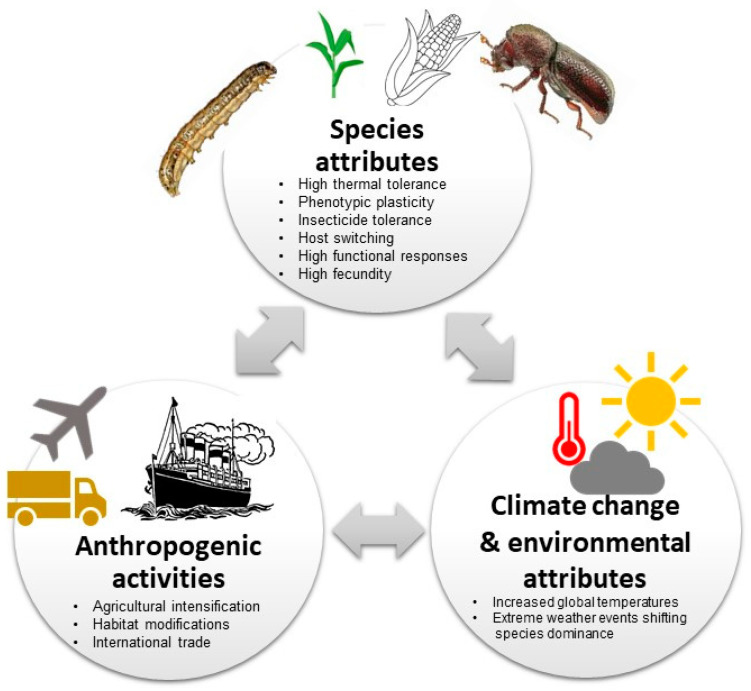
Summary illustration on the potential drivers of *Spodoptera frugiperda* and *Prostephanus truncatus* biological invasion in Africa (see also Nyamukondiwa et al. [14]).

Increasing agricultural intensification, international trade of agricultural products (see Figure 3), habitat modifications, anthropogenic climate change and the rise in human population size has led to a surge in invasive pest species, especially in tropical and subtropical environments [12,14,114,115]. Furthermore, land use and land cover changes (e.g., forest clearing for agriculture or pastureland, urban expansion or field abandonment) have played key roles in the introduction, establishment and proliferation of invasive species as they contribute to ecosystem disturbance (e.g., fragmentation), thus creating dispersal corridors [116,117]. Human modification of environments to optimise crop production through tillage and mineral nutrient application increases the nutrients and biomass of cultivated crops making them more attractive to pests than the surrounding vegetation [92]. Similarly, agricultural practices, e.g., irrigation also creates conducive microhabitats with limited thermal and desiccation stress, likely modifying the invasion ranges [118,119]. Additionally, human dietary shifts to fruits and vegetables and smallholder-based farming systems result in highly diverse agricultural ecosystems, which provide resource opportunities for polyphagous pests [120]. Similarly, the mixed cropping and grain and tuber (cassava) storage systems by smallholder farmers make host switches by *P. truncatus* highly inevitable. Moreover, using host wood and thatch as construction materials for storage structures complicates management options for *P. truncatus* and increases its potential for establishment in new areas [57]. As *S. frugiperda* and *P. truncatus* are both polyphagous, multiple cropping in most smallholder farming systems might have provided continuous food and winter habitats for the pests, providing niche resources to sustain populations and thus creating resilient bridgeheads to greatly extending their populations’ geographic range and temporal distribution [120]. The prevalence of maize and other *S. frugiperda* host plants, see [41], associated with suitable agroecological conditions in most of the regions, makes it a serious (and most certainly perennial) threat to food security in Africa [42,121].

#### 3.3.2. Climate Change and Environmental Attributes

Global increases in mean temperatures and changes in precipitation patterns due to climate change, coupled with the anthropogenic pathways described previously, have intensified the biological invasions of pest insects [26,122,123,124]. Climate change has been reported to influence the distribution and abundance of invasive insects both directly (e.g., by altering where species and hosts can occur) and indirectly (e.g., via changes in population growth rates, propagule pressure, and spread), among other factors [77,125,126,127]. Recent evidence shows poleward shifts in species for more benign environments as climate warming persists [126]. As global mean temperatures and variability increases, the threat of invasive insect species will increase as tropical and subtropical insects expand their range into more temperate areas [128,129]. Extreme weather events also promote invasive pathways through the modification of species hierarchies across tropical ecosystems, resulting in shifting species dominance and invasions [14]. These shifts are likely to modify competitive interactions, resulting in native communities that are more or less susceptible to colonisation by new invaders or expansion by established invaders [130]. Such changes to the bio-physical environments may result in changes in the abundance and geographic distribution of invasive species [131,132].

Temperature forms the first abiotic ‘ecological filter’ for successful invasion and establishment [133,134]. Some successful non-indigenous species are more tolerant to environmental and anthropogenic stressors than related native species, possibly stemming from evolutionary selection pressure (i.e., survival of only pre-adapted individuals for particular environmental conditions) during the invasion process [135]. Owing to this is the notion that invasive alien species are more eurythermal, i.e., able to maintain physiological functionality across variable temperatures [136,137]. Rapid adaptation is recognised as an important component of successful invasions [138]. Phenotypic plasticity can be adaptive and has been reported to improve survival in both Lepidoptera [139,140,141] and Coleoptera [142,143]. Desiccation stress, commonly associated with arid environments is one of the primary stressors influencing the distribution and behaviour of insects in the tropics [144]. Thus, as arthropods move from more mesic to xeric environments, they are faced with stressful desiccating environments [129,145]. Given the relationships between desiccation stress, temperature stress and other life history traits in arid ecosystems [144], the assessed desiccation tolerance in *S. frugiperda* in different developmental stages showed no negative impact on *S. frugiperda* fecundity following exposure to desiccation pre-treatment. This desiccation resistance may have aided in the species survival and ultimate success in arid and semi-arid environments [146] as this contributed to the unabated perpetual reproduction and fitness of the moth species under stressful arid environments.

The direct effects of evolutionary history, behaviour and physiology on the ecology and species biological responses to rising global temperatures are increasingly being documented [137,147,148]. Environmental conditions can alter the form, function and behaviour of organisms through physiological responses over short and long timescales and even over generations [149,150]. In order for invaders to become established in a recipient environment, they must first pass through the ‘ecological filter’ of that environment [14,133]. The ecological filter is composed of two overarching components, the biotic and the abiotic [133,151]. Biotic factors include the ability to compete with native species for both resources and niche possession and avoiding predation by local opportunistic predators [152,153]. Temperature and relative humidity are the most important abiotic factors faced by invaders in new regions [14,129,136,142,154]. They require the insect to adjust its physiological responses to adapt to prevailing conditions [14,149,151]. Failure to overcome both biotic and abiotic factors can prevent establishment or further range expansion.

Invasion success is also affected by intrinsic attributes of species and characteristics of the invaded habitat [155]. Tropical climates typical in Africa are characterised by extreme weather events such as high temperatures and seasonal droughts, thus, for successful invasion, insect pests have to adapt to these extreme climate features [14]. These climatic and weather changes not only affect the status of insect pests but also affect their population dynamics, distribution, abundance, intensity and feeding behaviour [77,156,157,158]. In Africa the highest densities of *P. truncatus* tend to occur in humid lowlands, in contrast to meso-America where the pest tends to occur in greatest numbers in cooler upland regions [159]. Arthur et al. [63] conducted a predictive model that found *P. truncatus* has been limited to tropical and subtropical regions but could likely spread to temperate regions as temperatures rise with climate change. On the other hand, African climate is conducive for *S. frugiperda* proliferation as the pest originates from tropical and subtropical South America with a largely similar climate to tropical Africa [40,42].

Climate change, particularly increasing temperatures, have both direct and indirect effects on insect development and survival. Firstly, climate change may have adverse effects on the activity and effectiveness of natural enemies through top-down effects [160]. The organisms most affected by increasing temperatures are higher trophic levels, including natural enemies (e.g., predators and parasitoids), and this may affect their efficacy as biological control agents reviewed in [160]. While both *S. frugiperda* and *P. truncatus* are known to have high thermal tolerance [14,124,161,162], the abundance and efficacy of natural enemies can be negatively affected at higher temperatures as higher trophic levels are affected more disproportionally than lower trophic levels [163], affecting antagonism and leading to invasive species proliferation. The rampant spread of especially *P. truncatus* and to a lesser extent *S. frugiperda* across Africa has been hypothesised to be largely aided by the lack of adapted natural enemies during the early stages of invasion, see [53,164], and thus greater losses have been reported.

Climate change also alters the interactions between the insect pests and their host plants. It also influences the range and quality of host species through interaction with edaphic conditions and nutrient supply status of host plants, thereby indirectly affecting their life history traits and survival chances. For instance, elevated temperatures increase the concentrations of plant secondary metabolites, particularly condensed tannins and total phenolics, which ultimately influence the thermal tolerance parameters of herbivorous insects that feed on them [125]. For *S. frugiperda*, the effects of diet and temperature have been well documented by [165]. The rate of insect multiplication might also increase with an increase in carbon dioxide (CO_2_) and temperature, owing to the bottom-up effects associated with an increase in, e.g., plant host growth under optimal high CO_2_ and temperature environments. Similarly, large scale changes in rainfall associated with changing climates will have a major effect on the abundance and diversity of arthropods [166].

#### 3.3.3. Species and Event Attributes Leading to Biological Invasions in Africa

##### Shared Attributes across Aggressive Invaders

Invasion success by IAS is not only influenced by the characteristics of the invaded habitat, e.g., agroecology; the intrinsic attributes of invasive species also have a significant contribution [155]. Common shared attributes across aggressive invaders have been summarised by [14] and include high basal thermal tolerance, phenotypic plasticity, desiccation tolerance, insecticide resistance, host switching, high functional responses, high propagule pressure, integrated stress resistance and others (also see [117,145,151]). On the contrary, native species usually have lower competitive ability; lower dispersal abilities and reproductive edge [167,168]. While these characteristics vary across taxonomic groups, notable trait overlaps are common across the most prolific insect pest invaders [169,170]. For example, generalist predatory habits [171], dynamic population growth after an initial lag period [169] and superior competitive ability relative to native organisms [172] are among several of the components that have been shown to facilitate the establishment of non-native species [151].

##### Species and Event Attributes of *Spodoptera frugiperda*

In *S. frugiperda*, the adult insect can self-disperse by flying over long distances to new environments. High reproduction, shorter life cycles, no diapause and host plant switches allow species to thrive in diverse environments [39,41]. Notably, the most prolific invasive species can feed on broad diets, i.e., polyphagous [173,174]. This is particularly important during species introduction and ensures survival in new areas. For *S. frugiperda*, its ability to feed on many hosts (~353 plants species from 76 families), mainly from the Poaceae, Asteraceae and Fabaceae families [41], presents the pest with excellent host-switching opportunities. Maize is the preferred host plant. However, in its absence, the pest can survive on sorghum (*Sorghum bicolor* L. Moench), cotton (*Gossypium hirsutum* L.), wheat (*Triticum aestivum* L.), cabbage (*Brassica oleracea* L.), cassava (*Manihot esculenta* Crantz) tomato (*Solanum lycopersicum* L.), beans (*Phaseolus vulgaris* L.), cowpea (*Vigna unguiculata* L. walp.) [175], banana (*Musa nana* Lour.) [176] and other wild hosts; see also [41,165]. However, in Africa, *S. frugiperda* has been primarily reported to infest maize followed by sorghum [175,177].

Due to their high fecundity, insects are more likely to survive and spread to newly introduced environments [102]. Following successful invasion, ecological, economic and human health issues arise as a result of the establishment of IAS. The history of a species in its native range is a good predictor of potential impacts in the introduced environment [93]. The high fecundity of *S. frugiperda* and its ability to migrate long distances are two of the species’ traits that could also explain the speed at which it invaded the continent [42,121]. Exceptionally high fecundity allows for the rapid establishment of a species post-invasion [44,178]. In addition, adults have been known to migrate several hundreds of kilometres [179,180]. The adult moths can fly continuously for over 24 h and cover over 400 km through self-powered flight [181]. In terms of larval dispersal via ballooning, *S. frugiperda* was found to have a wider dispersal and plant damage potential than any of the indigenous stemborer species *Busseola fusca* (Lepidoptera: Noctuidae) and *Sesamia calamistis* (Lepidoptera: Noctuidae) [182].

The high supply and frequency of propagule introductions might have increased the chance of successful invasion due to high genetic diversity, continual supplementation and increased probability of finding host plants and introduction to a favourable environment [183,184,185]. The invasion success of *S. frugiperda* has been attributed to high parental propagules and multivoltine nature [14,124,182,186]. In addition to the high genetic diversity found in this species, human-assisted long-distance movements can reciprocate introductions of genotypes from invasive populations to native populations [180]. *Spodoptera frugiperda* can feed on any part of the host plant, e.g., on leaves, tassels and ears on or before the soft dough stage. In addition, the insect has a relatively shorter lifespan (3–4 weeks) and can adjust the number of larval instars depending on diet [165] compared to related species, e.g., stem borers. This enables it to complete several generations per season and quickly develop insecticide resistance mechanisms as well as evading unfavourable habitats. Successful management of *S. frugiperda* has historically relied upon application of synthetic insecticides and through cultivation of genetically engineered crops expressing insecticidal proteins (Bt crops) [187,188]. *Spodoptera frugiperda* has, however, developed resistance to both synthetic insecticides (e.g., organophosphates, carbamates, pyrethroids and diamides) and Bt crops, which risks undermining the benefits delivered by these important crop protection tools [188]. Also, the cryptic feeding behaviour of larvae can further limit pesticide effectiveness [189]. For *S. frugiperda*, there are up to 150 parasitoid species, with a large number of them (80 species) originating from South America [47,190]. These parasitoids include *Telenomus remus* (Nixon), *Meteorus* sp., *Chelonus texanus* (Cresson), *Cotesia marginiventris* (Cresson) and *Aleiodes* sp. [190,191] in the Americas. In Africa, over 30 *S. frugiperda* parasitoids have been identified *viz Coccygidium luteum* (Brullé), *Trichogramma* sp., *Telenomus* sp., *Drino quadrizonulla* (Thomson, 1869), *Metopius* cf. *discolor* (Tosquinet), *Charops* sp., *Cotesia icipe* (Fernandez and Fiaboe) and *Palexorista zonata* (Curran) [47]. Despite the availability of these natural enemies, *S. frugiperda* damage remains serious in Africa due to the overuse of pesticides in agroecosystems that compromise the field efficacy of these biological antagonists coupled with environmental conditions permitting the moth’s all-year round development.

##### Species and Event Attributes of *Prostephanus truncatus*

*Prostephanus truncatus*, though not as devastating in its native range in central America as it is in invaded areas [104], causes much higher damage in the introduced regions in Africa primarily due to the lack of and/or failure of its natural enemies [103]. As such, unregulated populations result in the wide dispersal and fast colonisation of hosts leading to high losses in stored maize and dried cassava roots [29]. As previously alluded to, at the time of the accidental introduction of *P. truncatus* in Tanzania, there were no suitable pesticides registered for control of the pest as it required organophosphate–pyrethroid combinations rather than just organophosphates which could effectively control all other storage insect pests [109]. Competition is one of the key elements propelling invasive species [192]. Although some studies have found *Sitophilus zeamais* Motschulsky (Coleoptera: Curculionidae) to be the better competitor compared to *P. truncatus* [192], most agree that the latter fares much better in conditions found in most storage facilities, i.e., high temperature and low relative humidity and has a competitive advantage as an invasive species in new areas with stored maize, even in the presence of *Sitophilus oryzae* (L.) (Coleoptera: Curculionidae) [192,193,194,195]. However, most of these studies were laboratory-based and therefore more investigation is required on the competition phenomenon as this could play out differently in nature or under simulation. Pesticide tolerance also adds to the superior attributes of *P. truncatus*. While neonicotinoids have proven efficacious against *P. truncatus* [37,196], there has been evidence of tolerance to organophosphate and pyrethroid formulations [30,197,198]. *Prostephanus truncatus* also produces copious amounts of grain dust which dilutes the applied pesticides; thus affecting pesticide efficacy [37,199]. The increased rates of pesticide degradation due to increasing temperatures [37], coupled with *P. truncatus*’s high thermal tolerance and insecticide resistance mechanisms enhances the chances of survival of the pest over other species sharing the same ecological niche with it. Studies have also shown that apart from maize, *P. truncatus* can breed on a wide range of other plant substrates (branches, roots and seeds), has adapted to alternate hosts, e.g., cassava, and can persist in non-agricultural habitats [57,63,159]. Muatinte and Berg [200] listed 13 trees and 8 grass species on which *P. truncatus* bred and survived on in the wild. The tree species include *Brachystegia spiciformis* Benth, *Colophospermum mopane* (Kirk ex Benth.) and *Strychnos spinosa* Lam, and fresh and dry grass stems of species, including *Pennisetum glaucum* (L.) R. Br, *Hyparrhenia hirta* (L.) Stapf, *Acroceras macrum* Stapf, *Digitaria eriantha* Steud and *Aristida congesta* Roem and Schult [200,201]. The beetle possesses α-amylase and proteases which aid in the digestion of a wide variety of diets, including hard woody material [202,203,204]. The species attributes of *S. frugiperda* and *P. truncatus* that aid their invasiveness are summarised in Table 1.

## 4. Impacts of *S. frugiperda* and *P. truncatus* Biological Invasions

### 4.1. Overview

Invasive alien species have environmental, economic and social impacts, disproportionally threatening the livelihood and food security of smallholder farmers in low- and medium-income countries [9,10,11,205]. In most cases, farmers and governments often invest huge sums of money towards synthetic pesticides, the major and first control option used against invading pests [39,40], seldom trading off other important sectors, e.g., healthcare and education [206,207]. Given pesticide resistance, many of these insecticides are often ineffective [188]. Furthermore, resource-poor farmers in developing countries usually cannot afford personal protective equipment and lack the knowledge and understanding of chemical pesticides and their safe use [46], which compromises their proper use and risks exposure to toxic substances, resulting in accidental poisonings. Widespread and indiscriminate use of chemical pesticides also undermine environmental quality (biodiversity loss and pollution of air and water) and the pest control services provided by natural enemies [208].

### 4.2. Economic Costs of S. frugiperda and P. truncatus Invasions

The impacts of *S. frugiperda* and *P. truncatus* can be defined and quantified as economic costs, i.e., expenditures to prevent, reduce or alleviate the losses caused by these pests [21] or the marketing losses resulting from compromised quality. In Africa, IAS generally can cause up to a 35% loss in national gross domestic product (GDP) [209]. Severe maize infestation by *S. frugiperda* can reduce per capita household income by 44% and increase a household’s likelihood of experiencing hunger by 17% [44]. Infestation by *S. frugiperda* reduces maize yields by up to 54% [32,42,210] and can cause up to USD 13 billion per annum crop losses across Africa [42]. Various reports have recorded even higher estimated losses per annum see [44,211]. In Ghana and Zambia, the annual loss estimates for 2017 were USD 177 million and USD 159 million, respectively [44]. In Ethiopia, the pest caused an average annual loss of 36% in maize production, reducing yield by 0.225 million tonnes of grain between 2017 and 2019 [33]. In Kenya, *S. frugiperda* caused losses of approximately 33% of the annual maize production, estimated at approximately 1 million tonnes, with large variations across regions [32,47]. Rwomushana et al. [44] extrapolated that the pest had the potential to cause an annual reduction in maize production in Zimbabwe of approximately 264,000 tonnes, translating into revenue loss of USD 83 million. More costs related to *S. frugiperda* damage are highlighted in Table 2.

On the other hand, grain damage due to *P. truncatus* can level up to 100% and weight losses between 30 and 50% have been reported in stored maize [24,36,37,38]. Costs related to damage and losses as well as the costs of controlling *P. truncatus* in maize are scarce primarily because the costs cannot be isolated from those of co-occurring pests such as *S. zeamais* and *Tribolium* spp. When *S. frugiperda* and *P. truncatus* occur in the same environment, they have the potential to further disrupt vulnerable Africa’s food systems through synergistic interactions. Invasive species also comprise one of the most apparent risks of the globalisation of international trade to both agricultural and related products [19]. This is because IAS can disrupt trade across countries, particularly in developing African regions, where phytosanitary measures are relaxed and ineffective [108]. When the losses caused by the *P. truncatus* became more apparent in the literature, many African countries declared it a quarantine pest and prohibited the importation of maize from infested countries or after transit through these countries [212]. This approach, however justified at that time, not only caused a loss of export markets to African countries that had a surplus of maize (In particular, Tanzania), but also complicated logistics and increased the costs of the provision of ‘relief maize’ by the international community after the drought in southern Africa in 1991/1992 [213,214]. Combined field and postharvest losses due to *S. frugiperda* and *P. truncatus* led to food shortages by removing part of supply from the market, thus contributing to high food prices [193].

### 4.3. Direct and Indirect Effects of S. frugiperda and P. truncatus on Human Health and Nutrition

Economic losses experienced when invasive species affect food production also result in negative effects on human health, directly or indirectly. By contributing to huge losses in maize, both *S. frugiperda* and *P. truncatus* contribute to malnutrition negatively affecting the health of many people across the continent. Tambo et al. [205] found that households affected by *S. frugiperda* were 12% more likely to experience hunger, as measured by the household hunger scale. Farm losses incurred have cascading effects of reducing agricultural production, which is largely menial in Africa [23], thus further compounding food insecurity challenges. Human health is also affected by product contamination in storage, i.e., infestation by *P. truncatus* can increase the moisture content of the stored grains, inadvertently creating a favourable environment for fungal growth, e.g., *Aspergillus flavus* which can produce some carcinogenic aflatoxins in food products [193]. Furthermore, insect feeding also causes nutritional postharvest losses reducing basic access to nutritious food for consumers [17,215]. Cereal grains comprise 30–60% of the daily caloric intake for humans around the globe [216]. Maize, for instance, is central to food and nutrition security for millions of people in Africa, which consists of 54 countries populated by over one billion people and accounts for 73% of the calorific intake within the region [217,218,219,220]. The consumption of insect-damaged grain which potentially has low nutritional value exposes the population to malnutrition [11].

The initial detection of *S. frugiperda* and *P. truncatus* is usually followed by the haphazard use of pesticides, leading also to increased human exposure to pesticides. For example, in 2017, Zimbabwe distributed nearly 102,000 L of pesticide valued at USD 1.97 million to farmers [40]. The continuous and injudicious use of these chemical insecticides poses adverse risks to human and environmental health, including the loss of biodiversity, e.g., natural enemies and pollinators [11,220]. This also increases the costs incurred in mitigating and managing the pest, a feat that is often difficult for resource constrained African farmers [136,221].

### 4.4. Ecological Costs of Biological Invasions

Biological invasions rank among the most significant threats to biodiversity and ecosystems and are considered the second most serious cause of species extinctions [222,223]. Their ecological impacts can be so severe that they are considered as one of the major drivers of biodiversity loss across the globe [12,224,225]. They are associated with an average of a 25% decline in native species diversity, and increasing abundances of non-native predators are linked to a 44% decline in native species population [226]. Indeed, the impact of invasion by a single non-native species on the function and structure of ecological communities can be devastating as they have detrimental effects on ecosystem functioning and the delivery of ecosystem services [4,12,13]. The interactions among species in an ecological community can be significantly altered as the introduction of an exotic species can influence species composition, richness and abundance; thereby disrupting the structure of local food webs and patterns of interspecific interactions [3,4]. Using data from InvaCost, a repository of costs of invasive alien species [13], estimated the cumulative cost of biological invasions in Africa to a range between USD 18.2 billion and USD 78.9 billion for the period from 1970 to 2020. Worryingly, the reported costs are mostly associated with the damage caused by invasive alien species without considering those of controlling the incursions. Consequently, the actual total costs were grossly under-estimated. The majority of reported costs are, however, skewed towards the agriculture and health sectors, which are considered economic activities compared to ecosystem services [21].

Field studies conducted in Uganda revealed that the invasion by *S. frugiperda* has caused the decline of stemborer incidences in maize and the displacement from the maize crop, as their preferred host plant, to sorghum [177,220]. There is interspecific competition among these species at the larval stage in the utilisation of maize—the preferred host [182,227]. Such interactions are likely to influence community structure of these lepidopteran herbivores in areas where they co-exist [220]. Introduction of species into new environments can trigger rapid evolution, for example, functional responses, and thus increasing the damage potential of alien invasive species [228]. Furthermore, multiple introductions of species from different biogeographical regions can result in cryptic interactions leading to admixture of genetic characteristics leading to changes in genomic structure of the IAS [101,228,229]. Rane et al. [230], for example, associated multiple *S. frugiperda* introductions into Asia and Australia with genetic hybridisation, backcrossing and genome doubling, see also [209], linking these with the introduction of insecticide resistance alleles in established populations. Such genetic hybridisation complicates pest management, leading to increased crop losses.

Similarly, studies have shown that invasive species that occur in postharvest agricultural commodities are often more competitive and can overcome competition and even displace other native species [192,195,231,232]. Quellhorst et al. [195] examined the competition between *S. zeamais* and *P. truncatus* on maize at four varying temperatures and found that increasing temperature resulted in elevated population growth of the invasive *P. truncatus* at the expense of *S. zeamais*. Other impacts noted included direct competition, changes to ecosystem functioning, hybridisation and predation. Phylogenetic studies by [233] revealed significant additive genetic and environmental effects enhancing some traits (e.g., body weight) in strains of *P. truncatus* from different geographical locations, increasing fitness and thus invasiveness in certain populations. Similarly, genetic diversity in *T. nigrescens* characterised by allele insertions and deletions at specific loci may explain the variable success of biological control of *P. truncatus* with predators from different geographical locations [234]. Ecosystem dynamics are altered through a variety of interacting, mutually reinforcing mechanistic pathways, for example, species’ resource acquisition traits; population densities and the ability to engineer changes to physical environmental conditions [3]. Impacts to the environment such as pollution and development of pesticide resistance in pests arise through excessive and/or overapplication of synthetic pesticides in response to biological invasions [108]. This has negative implications on ecological services as they can lead to death of non-target organisms, e.g., pollinators, predators and parasitoids [235].

## 5. Management Strategies for *S. frugiperda* and *P. truncatus* Biological Invasions

### 5.1. Overview

Management of biological invasions can be divided into two stages: first, prevention through quarantine measures, and second, management through curative measures, which is a reaction to invasion following the detection of ecological impacts [236]. Usually, preventive measures are the first line of defence and if the results are unsatisfactory, curative measures are employed. In practice, the management of invasive species requires the application of a combination of these approaches.

### 5.2. Prevention through Quarantine Measures

Investment in biosecurity measures is important in monitoring and preventing introductions [237]. However, [238] noted that the unpredictable nature of potential invasion makes preventive management ‘riskier’ than control after establishment. The use of numerical trajectory models to predict the long-distance migration and possible destinations of insect pests is one example that can be used to monitor and detect invasions at early stages in areas under invasion risk [101]. However, it is practically impossible to detect insect pests at the initial infestation site at a sufficiently early stage to have chances of eradicating the pest [239]. Given that zero tolerance quarantine protocols require sampling every unit of imported goods, the default strategy therefore is to set acceptable tolerance limits (supported by technical information) for each pest sampled. In Africa, Salama and Abd-Elgawad [239] presented a table to determine the probabilities of detecting pest infestation levels when increasing numbers of samples are collected from an imported lot. Such a technique reduces labour, time and money and ensures certainty in the detection process. Budgetary constraints and bureaucracy, on the other hand, also tempt decision makers to intervene in the late-stage management of invasions [238]. In most invaded countries, therefore, the management of *S. frugiperda* and *P. truncatus* is limited to eradication strategies following invasion and the initial spread of the pests.

### 5.3. Curative Measures

The use of synthetic pesticides to control both field and storage insect pests is dominant in Africa [240]. Regarding *S. frugiperda*, control is maintained mainly through the use of a combination of synthetic pesticides and cultural (early planting, varietal selection and field hygiene) and mechanical methods [40,46,113]. Since the invasion of the African continent by *S. frugiperda*, huge quantities of pesticides amounting to trillions of US dollars have been used to control the pest ([241]; Table 2). However, the use of synthetic pesticides is unsustainable due to high costs, resistance development, pest resurgence and negative effects to environmental and human health [40,46]. This calls for the development and use of alternative control options, for example, systemic seed treatments [242]. Cultural control options, such as varietal selection, are key as the first line of defence against *S. frugiperda* and other pests. Host plant resistance is one of those methods that can be useful for *S. frugiperda* control [47]. There is thus need to identify and target those hosts/varieties for pest management. Crops grown under rainfed and mixed cropping systems were found to be less prone to attack by *S. frugiperda*, as rainwater tended to wash larval instars away [220]. This can be complemented by tillage systems where conventional tillage and frequent weeding was found to reduce *S. frugiperda* incidences through the exposure of pupae to the soil surface, thereby exposing them to the direct sunlight and predation [121,220]. On the other hand, intercropping with pumpkins was found to increase damage from *S. frugiperda* [121]. Mechanical and physical control methods are recommended under small-scale farming systems, as these methods are more practical on small pieces of land. These methods include handpicking and crushing the larvae and egg masses, and/or adding ash, saw dust or sand in plant whorls to desiccate the insects [39]. In addition, intercropping with non-hosts, such as common bean, and push–pull strategies are being advocated for [39,40]. Host plant resistance through the cultivation of Bt crops has also been an option for the control of *S. frugiperda* [187,188]; however, reports suggest the pest has developed resistance to Bt maize [188].

On the other hand, conventional synthetic insecticidal dusts have not guaranteed protection of stored maize grain against *P. truncatus* damage [38,199,243,244]. Neonicotinoid-based pesticides have been quite effective compared to organophosphate and pyrethroid active ingredients [245]. The use of entomopathogenic fungi, such as *Beauveria bassiana*, has been reported to be effective in controlling *P. truncatus* infestation in stored maize, though it would require periodic re-treatment after every 4 weeks to maximise grain protection during prolonged storage [246]. Combinations of enhanced diatomaceous earths (DEs) and natural products such as spinosad or low dose pyrethroids have also been proven effective both in the laboratory and in small scale grain storage systems [247,248] but are not available on the market. Host plant resistance though the selection and use of resistant varieties can be integrated with other control methods, such as synthetic pesticides, to improve *P. truncatus* management [51]. Recent research has thus focused on hermetic storage technologies which have brought the much-needed improved protection of stored grain commodities in much of Africa [249,250,251,252], safeguarding food and nutrition security while simultaneously reassuring pesticide-free food [244]. Grain imports can also be phosphine-fumigated on-board to control all life stages of insect pests before destination arrival [253,254]. Apparently, the literature on the economic impact of postharvest interventions is scarce, more so with specific reference to *P. truncatus*. In a comprehensive scoping study by [255], only 12.5% of the 334 studies reviewed reported economic outcomes. This shows that more evidence is required in this area in future studies. A robust postharvest loss assessment system for Africa is provided by the African Postharvest Loss Information Systems (APHLIS) online platform [256,257]. The platform provides loss estimates for different cereal grains by country, year, postharvest stage and the causes of postharvest losses [258,259]. The platform is expanding to include nutritional and economic implications of postharvest weight losses [257].

To successfully regulate invasive species therefore, both quarantine and eradication measures through voluntary and enforced legislation are required [260]. The use of lists of quarantine species at border crossings to prevent the introduction of IAS should increase between counties [261]. Furthermore, coordination across countries that share IAS is important as well as synchronising their regulations to prevent local spread. Postharvest wise, investment in road systems, infrastructure and logistics for grain movement, storage and processing are essential to reducing losses [68]. Increased international trade agreements may offer an opportunity for individual nations to harmonise quarantine policies [100]. Comparison of the environmental conditions of native and introduced ranges is useful in determining the likelihood of an introduced species’ establishment and invasiveness in novel ranges [93]. Using climate data from the native range of *P. truncatus* [63] predicted that the beetle will likely spread and become more aggressive in southern Africa due to similar climatic conditions, particularly high temperatures, compared to those found in Mexico and Central America, where the pest originated. Similarly, numerical trajectory models have placed southern Europe at risk of invasion by *S. frugiperda* from Egypt [101]. Thus, through dynamic modelling of climate data and species spatial–temporal dynamics, and accounting for the lesser sensitivity of biological invaders relative to natives [262], models have become essential to the control of biological invasions [263].

## 6. Conclusions

Efforts to improve regional food security in Africa continue to be hampered by the increasing threats of pest invasions across the food value chain. Climate change and increased anthropogenic activities, including trade and landscape modifications for agricultural purposes, are some of the major drivers of biological invasions in Africa. Since its introduction into Africa in 2016, *S. frugiperda* has become the most devastating field pest of maize—a staple food across sub-Saharan Africa and similar regions of the world. Similarly, *P. truncatus* exacerbates these food losses along the maize grain value chain, and the interaction between the two pests through cumulative synergistic damage on the same crop has led to aggravated staple food losses. In the case of *P. truncatus*, further economic losses are incurred through the loss of goodwill in terms of trade between countries or the extra measures that have to be taken when importing grain from *P. truncatus*-infested countries. Ironically, concrete data on economic losses caused by *P. truncatus* are scanty; and hence need greater attention in future studies. Integrated pest management strategies are key to the management of the two invasive species at national level, while pest monitoring and phytosanitary compliance are key at regional and international level. The aggressive nature of the two invasive insect species, extensive damage and associated attributes leading to their superiority, offer insights to researchers and policymakers on issues relating to future research studies and legislation for the control of biological invasions and mitigating their economic, environmental and societal impacts. This information is vital for improving food and nutrition security nationally and continentally through increased yield and the reduction of postharvest losses. The maintenance of resilient and integral food systems in highly vulnerable regions like Africa, e.g., through reducing the introduction and/or impacts of invasive agricultural pests, is of paramount importance for the realisation of the United Nations Sustainable Development Goals.

## Figures and Tables

**Table 1:** Summarised superior species attributes possessed by the *Spodoptera frugiperda* and *Prostephanus truncatus* that potentially enable them to outcompete native species in invaded territories (also see Nyamukondiwa et al. [14] and Kelley [151]).

*S. frugiperda*	*P. truncatus*
High fecundity and absence of diapause.	High reproductive capacity.
Self-dispersal through adult flights and larval ballooning.	Self-dispersal by adult flight.
High tolerance to pesticides.	High basal heat tolerance.
Larval internal feeding and grass cover limits pesticides’ contact and efficacy.	Sporadic and temporal occurrence, making it difficult to control.
Wide host range.	Alternates between cultivated and wild hosts, making it difficult to control.
Have predatory habits, allowing it to devour competitors.	Possesses α-amylase enzyme to aid in digestion of hard material such as timber.
	Produce copious amounts of dust during feeding, thus diluting pesticides and reducing their efficacy.
	Feeds from inside kernels thus evading contact pesticides.

**Table 2:** Summary table showing the estimated costs related to *Spodoptera frugiperda* in some African countries. The costs are related to field damage, cost of control (including pesticides) and related. This list may not be exhaustive but represents significant data obtained at the time of writing.

Reported Loss/Costs (USD)	Year	Loss/Cost Description	Country	Reference
40.2 million * (134,000 tonnes maize)	2017	Field damage to crops, amount of food that can feed 1.1 million people.	Ethiopia	[43]
2.5–6.2 million (8.3–20.6 tonnes maize)	2022	Estimated yield losses.	12 African countries	[42,45]
3 million	2017	For pesticides and provision for replanting. Cost of pesticides per household was USD 14.20 without subsidies and USD 7.30 with subsidies.	Zambia	[44,45]
159 million	2018	Value of maize field losses.	Zambia	[45]
4 million		Procurement of plant protection products.	Ghana	[45]
177 million	2018	Value of maize field losses.	Ghana	[45]
$25.30	2017	The amount spent on pesticides per household for those without subsidies. For those who received subsidies, the cost was USD 13.30.	Ghana	[44]

* Using an average regional price of USD 300 per tonne of maize.

## Data Availability

The datasets obtained during and/or analysed during the current review are available from the corresponding author on reasonable request.
